# New Clayey Deposit and Their Potential as Raw Material for Red or Structured Ceramics: Technological Characterization

**DOI:** 10.3390/ma14247672

**Published:** 2021-12-12

**Authors:** Ana Rosa S. Assunção, Gricirene Sousa Correia, Nazaré do Socorro L. S. Vasconcelos, Aluísio Alves Cabral, Rômulo Simões Angélica, Fabiana Pereira da Costa, Romualdo Rodrigues Menezes, Gelmires de Araújo Neves, Alisson Mendes Rodrigues, José Manuel Rivas-Mercury

**Affiliations:** 1 Federal Institute of Education, Science and Technology of Maranhão, Av. Getúlio Vargas-04, Monte Castelo, São Luís 65025-001, Brazil; anademel15@gmail.com (A.R.S.A.); gricirene@gmail.com (G.S.C.); ndsocorro@ifma.edu.br (N.d.S.L.S.V.); acabraljr@ifma.edu.br (A.A.C.); fabianacosta485@gmail.com (J.M.R.-M.); 2 Graduate Program in Geology and Geochemistry, Institute of Geosciences, Federal University of Pará, Av. Augusto Correa-01, Guamá, Belém 66075-110, Brazil; angelica@ufpa.br; 3 Graduate Program in Materials Science and Engineering (PPG-CEMat), Federal University of Campina Grande, Av. Aprígio Veloso-882, Bodocongó, Campina Grande 58429-900, Brazil; fabiana.costa@estudante.ufcg.edu.br (F.P.d.C.); romualdo.menezes@ufcg.edu.br (R.R.M.); gelmires.neves@ufcg.edu.br (G.d.A.N.)

**Keywords:** raw clay, new deposit, characterization study, technological properties, ceramic bricks and ceramic tiles

## Abstract

Mineralogical and technological characterization of ceramic raw materials from a new deposit located at Caxias city, Maranhão State—Brazil, was accomplished to determine their potential as raw materials for the ceramics industry in northeastern Brazil. The ceramic raw materials were collected from three different locations on the site and characterized by X-ray fluorescence (XRF), X-ray diffraction (XRD), differential thermal analysis (DTA), and thermogravimetry (TG). The XRF analysis of the fraction < 2 μm revealed that most samples had SiO_2_ (35–51 wt%), Al_2_O_3_ (19–29 wt%), Fe_2_O_3_ (2–21 wt%), MgO (0.7 to 4.5 wt%) and K_2_O (0.9 to 5 wt%) as components. Quartz, kaolinite, illite, hematite and montmorillonite were the main mineral phases identified. DTA and TG analysis confirmed the mineral identification. The technological potential of the ceramic raw materials was investigated by: cation exchange capacity (CEC), plastic behavior (Atterberg Limits), linear shrinkage at 950 °C (LS_F_), flexural strength (FS), apparent porosity (AP), water absorption (WA) and bulk density (BD). The main experimental results—WA (9–17%), AP (19–31%), FS (2.0–23 MPa), and the Atterberg limits—indicated that the ceramic raw materials investigated have high potential to be used to develop mass for red or structured ceramics, such as bricks and roof tiles.

## 1. Introduction

The civil construction sector is of great importance in the leading world economies since it contributes a significant share in gross domestic product. Despite the economic crises, this sector continues to grow [1]. In Brazil, the construction sector represents about 3.9% of the gross domestic product, and it is estimated to grow by 4% in this sector in 2021 [2]. In this context, clay raw materials play an essential role because they are the basis for manufacturing various materials to meet the growing and demanding consumer market. Among the main products manufactured from clay raw materials, it is possible to highlight blocks for sealing and roof tiles (structural ceramics), sanitary ware, ceramic tiles, refractories, and electrical insulators [3,4].

There are approximately twelve thousand small and medium-sized ceramic industries in Brazil, whose production units are concentrated in the southeast (66%) and south (25%) regions, where the most oversized ceramic poles in the country are located. Although Northeast Brazil has a smaller market share (~9%), the industry is expanding, mainly structural ceramic and artistic ceramic products [5,6].

**Figure 1:**
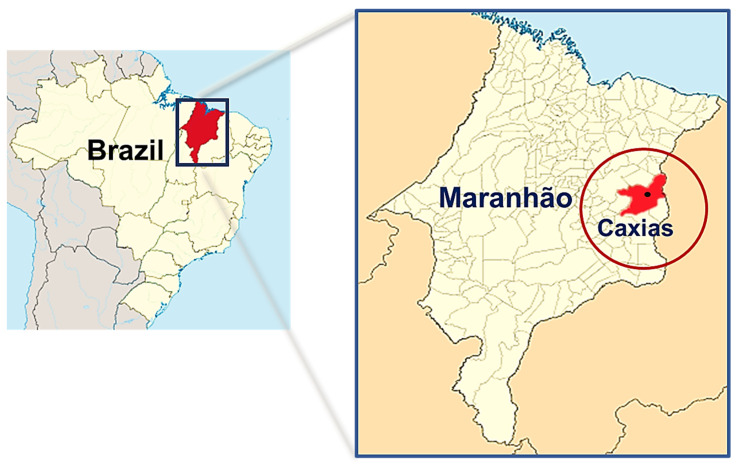
Location of the city of Caxias-MA, where the deposits investigated in this work are located (Source: Authors).

In the state of Maranhão (Brazil), where the investigated deposits are located, the ceramic sector is essentially composed of industries that operate in red or structural ceramics. According to data from the Brazilian Ceramic Association [6], there are approximately 120 companies in this industry segment in Maranhão, which yield about 31.2 × 10^6^ blocks/month and 18 × 10^6^ roof tiles/month and generate a lot of direct and indirect jobs. This industrial segment consumes 1.0 × 10^8^ tons/month of clay raw materials and invoices about $1.5 million per month, which shows the importance of the sector for the economy of this state. Figure 1 shows the Caxias—MA (Brazil) location, the site of the deposits investigated in this research.

It is worth mentioning that the manufacture of ceramic materials by most of these industries is still rudimentary, being produced through empirical mechanisms with low-quality products. Despite this, these industries supply a large part of the local consumer market [7]. Even so, the structural ceramic segment in Maranhão has new opportunities due to the expansion of the civil construction sector, which currently requires ceramic materials in greater quantity and better quality.

Given this scenario, discovering new clayey raw materials deposits with the technological potential to manufacture bricks and roof tiles is essential to expand Brazilian civil construction. In this context, this work investigates clays from newly discovered deposits located in the city of Caxias-MA (Brazil) to determine their potential as raw materials for the ceramics industry in northeastern Brazil. Clayey raw materials were characterized by physical, chemical, and mineralogical tests. The results obtained have the potential to contribute with technical and scientific information about the occurrence of clay minerals and improve the quality of ceramic products manufactured in this region.

## 2. Materials and Methods

### 2.1. Materials

**Figure 2:**
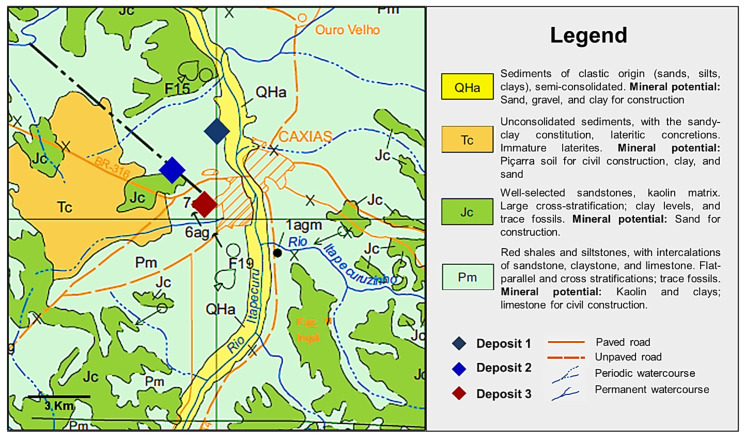
Geological map of the study area with the location of deposits studied highlighted (Adapted from topographic chart code SB.23-X-B, mapped by CPRM [8]). The rhombus black, blue, and red on the map represents the location of deposit 1 (Georeference—0680066/9464398), deposit 2 (Georeference—0678496/9432194), and deposit 3 (Georeference—0679716/9460542).

The clay raw materials investigated in this research were collected from three locations (deposit 1, deposit 2, and deposit 3) located in the Caxias city in the Maranhão state (Brazil). Figure 2 shows the geological map of the region where the studied clays were extracted. The rhombus on the map (black, blue, and red) represents the location of the three deposits studied. The area where the deposits are located is formed by red shales and siltstones, with intercalations of sandstones, claystones, and limestones, from the Motuca Formation. They are clay occurrences, as kaolinite, illite, and smectite in their composition. Quartz and feldspar minerals are also identified. In the Caxias sheet, a topographic chart mapped by the Brazilian Geological Service (CPRM) (code SB.23-X-B), the sedimentary structures in the deposits regions are from the Permian age [8].

**Figure 3:**
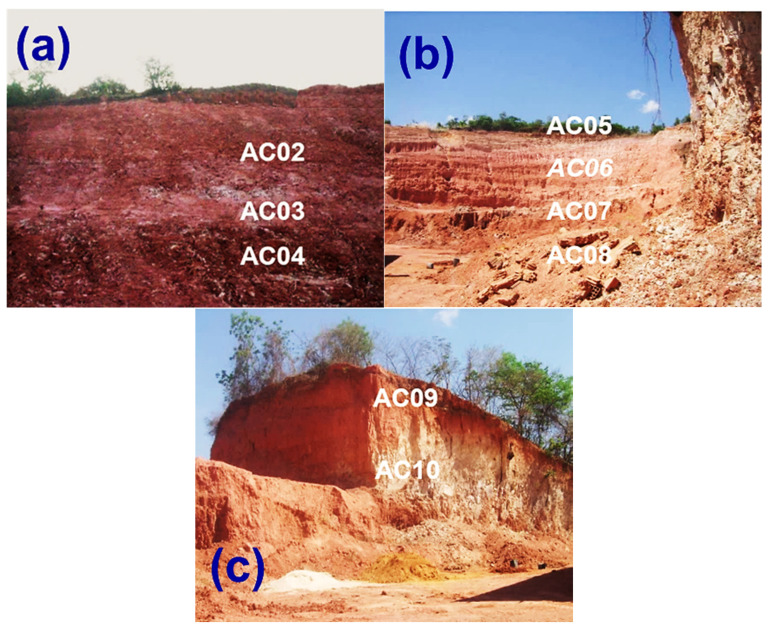
Outcrops of the deposits where the samples were collected: (**a**) AC02, AC03, and AC04 (deposit 1); (**b**) AC05, AC06, AC07, and AC08 (deposit 2); and (**c**) AC09 and AC10 (deposit 3).

In total, 9 samples were collected (see Figure 3a–c), where AC02, AC03, and AC04 from deposit 1 (Georeference—0680066/9464398), AC05, AC06, AC07, and AC08 from deposit 2 (Georeference—0678496/9432194), and, AC09 and AC10 were collected from deposit 3 (Georeference—0679716/9460542). Figure 3a shows the sampling scheme for samples AC02, AC03, and AC04 in deposit 1. The top of this deposit consists of a brown-colored material, a crust rich in granules characteristic of laterite. A reddish, silty-clayey and intensely kaolinized material is observed up to the foot of the hill. A reddish-colored material, silty-clayey, with the presence of mica and kaolinite, is observed from top to bottom in deposit 2 (Figure 3b). In deposit 3 (Figure 3c), there is a predominance of a silty-clay material of hard consistency and reddish color (top) interspersed with a whitish tone. This is indicative of kaolinite.

### 2.2. Methods

#### 2.2.1. Sample Preparation

##### Separation and Characterization of the Clay Fraction (<2 µm)

All samples were separated by sedimentation into fractions with particle size <2 μm from the pipette method. For this, ~50 g of the dry clay was added to an aqueous medium with 0.2 wt% ammonium hydroxide (NH_4_OH). The dispersion obtained was kept to stand for 24 h and later agitated for 2 h. Then, the dispersion was wet sieved over a 75 µm mesh and transferred to a 1 L beaker. At this time, the sedimentation test began, and the particle sizes were determined based on the Stokes law. The supernatant fraction was extracted (fine fraction), dried in an oven at 50 °C (24 h), and analyzed by differential thermal analysis and thermogravimetry (DTA/TG), X-ray diffraction (XRD), and X-ray fluorescence (XRF).

##### Drying, Grinding, and Quartering of the Samples for Technological Tests

The samples collected from deposits 1, 2, and 3 were sun-dried for 48 h and disintegrated with a rubber hammer. Then, the samples were inserted in an oven with renewal and forced air circulation and kept at 50 °C ± 2 °C for 24 h. After drying, the samples were comminuted in a disc mill, homogenized by hand, and subsequently quartered to provide a statistically significant number of samples. Finally, the samples were further pulverized in a motorized mortar mill (Marconi, model MA 890, Piracicaba, São Paulo state, Brazil) and sieved (<75 μm). The particle size distribution of the clay samples was determined by a wet sieving method after grinding and dispersion in an aqueous medium using 0.2 wt% of ammonium hydroxide (NH_4_OH) as the dispersant.

##### Preparation of the Specimens for Determination of Physicomechanical Properties

The specimens for physicomechanical tests were prepared from powder samples moistened at 8%, which were homogenized in a ball mill for 30 min and sieved (25 μm). Then, the samples were kept at rest for hours in a closed polyethylene bag. The powder was inserted into a stainless-steel mold and pressed uniaxially (25 MPa) to obtain specimens with dimensions of e 80 mm × 20 mm × 5 mm. The samples were sintered in an electric oven according to the following route: (i) heating (5 °C·min^−1^) from room temperature to 125 °C, where they were kept for 1 h; (ii) heating (5 °C·min^−1^) to 550 °C, with a dwelling time of 1 h; (iii) heating to 950 °C (5 °C·min^−1^), and maintained for 3 h; and, (iv) cooling (5 °C·min^−1^) to room temperature.

#### 2.2.2. Analytical Techniques and Methods

Chemical analyses were performed by X-ray Fluorescence Spectrometry (XRF) (Axios Minerals, PANalytical, Almero, The Netherlands) with a ceramic X-ray tube and 2.4 kW Rh anode, using 8 g of the lithium Tetraborate fondant (Li_2_B_4_O_7_) for 1 g sample. Data acquisition was made with the SuperQ Manager software, and the data were treated with the IQ + software, also from PANalytical (Almero, The Netherlands).

Loss on ignition (LOI) experiments were carried out in a muffle furnace. For this, 5 g of each sample was inserted into an alumina crucible and heated (5 °C·min^−1^) to 1100 °C, with a holding time of 1 h, then cooled to room temperature. LOI values were calculated according to Equation (1).

(1) $$\mathrm{LOI}=\frac{m_{d}-m_{f}}{m_{d}}\times 100\%$$

where *m_d_* is the mass of the sample dried at 110 °C (g), and *m_f_* is the mass of the sample after firing at 1100 °C (g).

The diffractograms of the coarse fraction (<75 µm) and clay fraction (<2 µm) were continuously recorded in an X’PERT-PRO X-ray diffractometer (PW 3040/60, PANalytical, Almero, The Netherlands) equipped with an X’Celerator detector (RTMS, PANalytical, Almero, The Netherlands), a PW3050/60 goniometer with theta–theta design; with a step size of 0.02° (2 θ) in the angular range of 5–70° (2 θ); using Cu–Kα radiation (λ = 1.540598 Å) at 40 kV and 40 mA. For the more accurate identification of the minerals present, the clay fraction (<2 µm) was also evaluated under oriented conditions, saturated with ethylene glycol, and after thermal treatment at 550 °C for 2 h. The analysis was performed in the angular range of 4–35° (2 θ). The XRD patterns were analyzed with High Score Plus 3.0 e software (PANalytical, Almero, The Netherlands). The crystalline phases were identified using the Powder Diffraction File—International Center for Diffraction Data (PDF-ICDD).

The differential thermal calorimetry (DTA) and thermogravimetry (TG) experiments were carried out on PL-Thermal Sciences equipment (Aldridge, UK) with a simultaneous thermal analyzer STA 1000/1500 Stanton Redcroft Ltd. (Aldridge, UK). The samples (≈18 mg) with particle size less than 2 µm were heated at 10 °C·min^−1^ from room temperature to 1100 °C in an alumina crucible and under a dynamic atmosphere of nitrogen (20 mL·min^−1^).

#### 2.2.3. Technological Tests

##### Cation Exchange Capacity (CEC)

The methylene blue staining method [9,10] was applied to assess the cation exchange capacity (CEC), according to ASTM C837-09 [11]. For this, 100 mL of distilled water was added to 1 g of clay samples (<75 μm) and taken separately to an ultrasound bath for 3 min. A solution of HNO_3_ (0.01 N) was used to adjust the pH of the suspension to 2.5–3.5. Equation (2) was used to calculate CEC values.

(2) $$\mathrm{CEC}=\frac{V\times C\times 100}{m}\left(\frac{meq}{100g}\right)$$

where *V* is the volume of methylene blue consumed (mL), *C* is the concentration of methylene blue (N), and *m* is the dry clay mass (g).

##### Plastic Behavior

Plasticity tests were performed to determine the Atterberg limits (plasticity limit (PL), liquidity limit (LL), and plasticity index (PI)). These parameters indicate the water content (%) added to the ceramic mass to achieve a certain consistency. Plasticity is one of the most critical parameters for the manufacture of clay products [12]. The tests were carried out in a Forteste electric device model Casagrande electric with counting strokes, following the recommendations of the standard ASTM D4318 [13].

##### Physicomechanical Properties

Immediately after sintering, the specimens (80 mm × 20 mm × 5 mm) were placed in a desiccator. The following physical-mechanical properties were evaluated: linear shrinkage after firing (LS_F_), 3-point flexural strength (FS), water absorption (WA), apparent porosity (AP), and bulk density (BD). The LS_F_ values were determined from Equation (3).

(3) $${\mathrm{LS}}_{F}\left(\%\right)=\frac{L_{i}-L_{f}}{L_{i}}\times 100$$

where $L_{i}$ and $L_{f}$ are the lengths of the specimens before and after firing.

The three-point flexural strength tests were performed on a universal TIRATEST 2000 machine with a loading speed (deformation) of 0.1 mm.min^−1^, following the standard ASTM C674-13 [14]. Specimens with dimensions of 80 mm × 20 mm × 5 mm were used in the experiments, and the FS values were calculated from Equation (4).

(4) $$\mathrm{FS}=\frac{3\times F\times L}{2\times b\times h^{2}}$$

where *F* (N) is the load reached the moment of rupture; *L* (mm) is the distance between the two supports, *b* and *h*, the width and thickness of the specimens (mm), respectively.

The Archimedes method was used to measure water absorption and apparent porosity. The experiments were carried out according to the ASTM C20-00 standard [15]. For this, specimens (80 mm × 20 mm × 5 mm) were weighed before and after water immersion (24 h). The weighing was carried out on a scale (BEL, model L303iH, Monza, Italy) with an accuracy of 0.001 g. Equations (5) and (6) were used to calculate the water absorption and apparent porosity, respectively.

(5) $$\mathrm{WA}\left(\%\right)=\frac{W_{w}-W_{d}}{W_{d}}\times 100$$

(6) $$\mathrm{AP}\left(\%\right)=\frac{W_{w}-W_{d}}{W_{w}-W_{i}}\times 100$$

where $W_{w}$, $W_{d}$, and $W_{i}$ are the weights of the specimens, wet, dry, and immersed, respectively.

The determine the bulk density, the experimental procedure adopted consisted of measuring the masses and dimensions of the specimens, which are measured after pressing (green body), drying at 110 °C, and firing at 950 °C [15]. BD values were calculated from Equation (7).

(7) $$\mathrm{BD}=\frac{m}{v}$$

where *m* (g) is the mass of the specimen and *v* (cm^3^) is the apparent volume.

## 3. Results and Discussion

### 3.1. Mineralogical, Chemical Compositions and Thermal Behavior of the Clay Raw Materials

**Figure 4:**
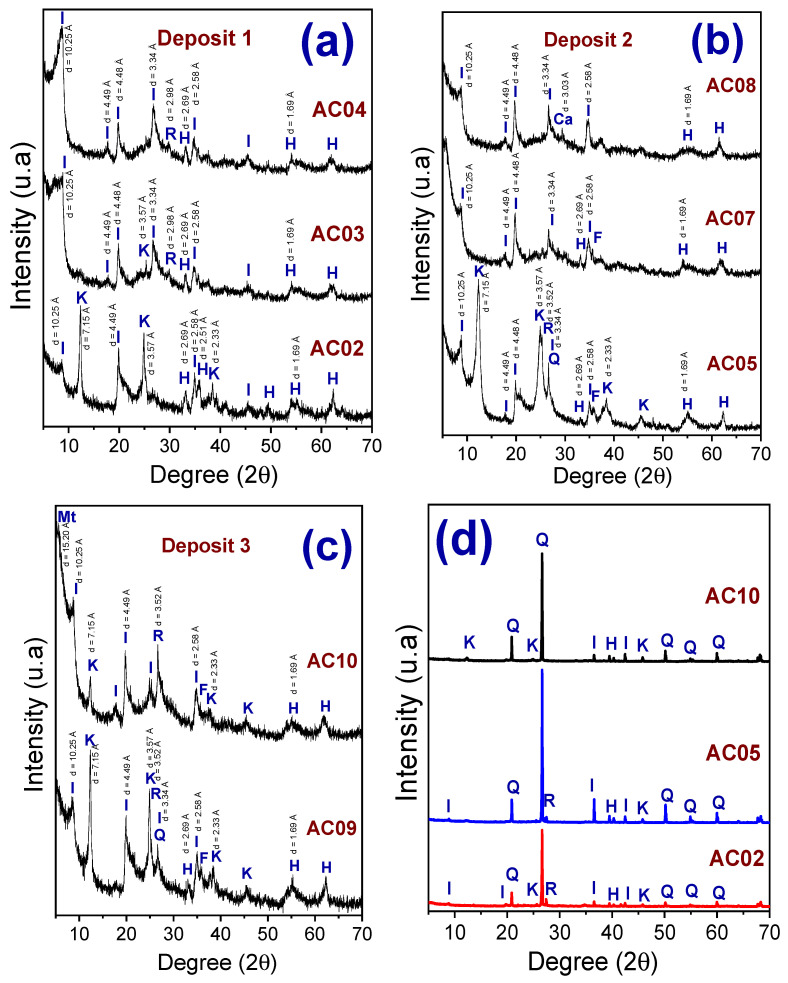
X-ray diffraction pattern of the samples (AC02, AC03, AC04) (**a**), (AC05, AC07, AC08) (**b**), and (AC09 and AC10) (**c**), clay fraction (<2 µm). Including the diffractograms of the samples AC02, AC05, and AC10, the coarse fraction (<75 µm) (**d**). (I—Illite, K—Kaolinite, Mt—Montmorillonite, R—Rutile, H—Hematite, Ca—Calcite, Q—Quartz, F—Feldspar).

Figure 4a–d shows the X-ray diffraction pattern of the clay fractions separated by sedimentation (<2 µm) (AC02, AC03, AC04, AC05, AC07, AC08, AC09, and AC10) (Figure 4a–c) and of the clay and silt fractions (<75 µm) (AC02, AC05, and AC10) (Figure 4d). The following mineralogical phases were identified in the fine fraction: kaolinite, illite, rutile, hematite, calcite, quartz, and feldspar, see Figure 4a–c. The presence of the illite explains the high concentration of K_2_O and MgO detected in the chemical analysis (Table 1a). A peak related to montmorillonite (2 θ = 5.81°; d = 15.20 Å) was detected in sample AC10. The X-ray diffraction pattern of coarse fraction (raw powder) (Figure 4d) showed the predominance of quartz in all clay samples. Kaolinite, mica/illite, rutile, hematite were also identified. All samples of the coarse fraction showed a similar X-ray diffraction pattern.

**Figure 5:**
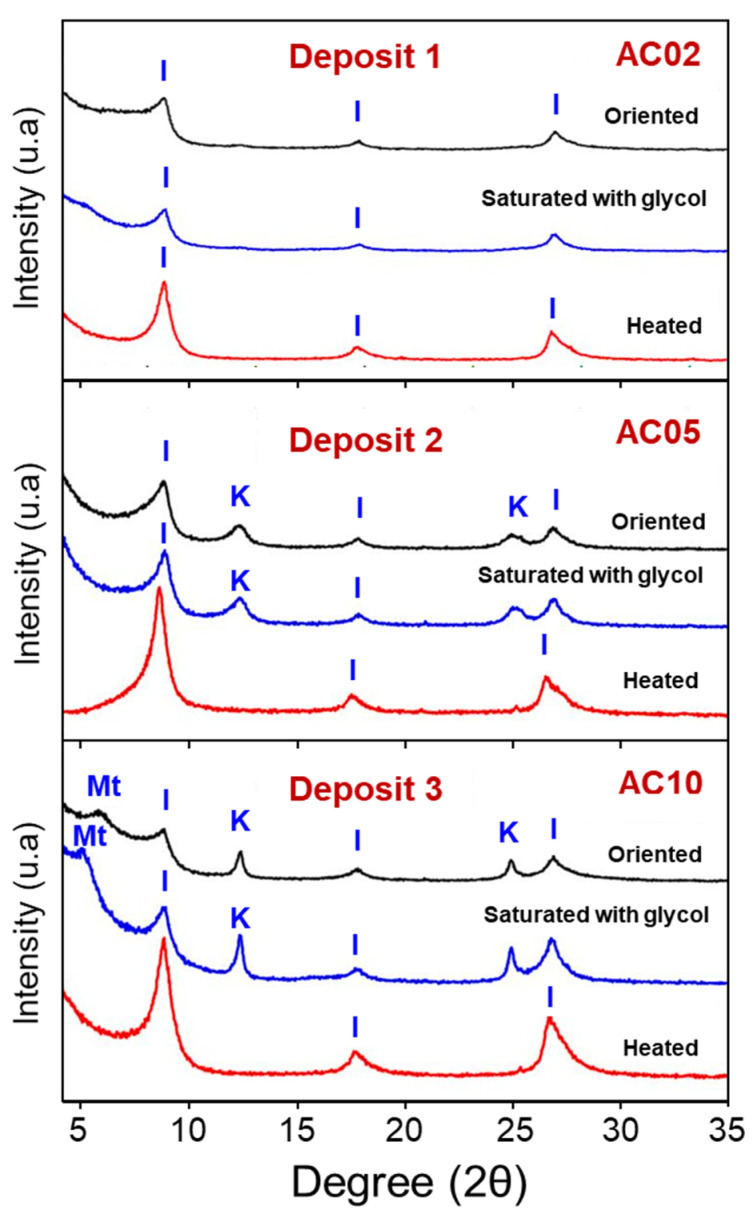
X-ray diffraction pattern of the samples AC04, AC05, and AC10, clay fraction (<2 µm) under oriented conditions, saturated with glycol and heated to 550 °C. (I—Illite, K—Kaolinite, Mt—Montmorillonite).

Figure 5 shows the X-ray diffraction pattern of the clay fractions separated by sedimentation (<2 µm) under the oriented conditions, saturated with glycol, and heated (samples AC04, AC05, and AC10). In samples AC04 and AC05, the presence of expansive clay minerals (montmorillonites) was not detected. AC04 showed a similar X-ray diffraction pattern under the three conditions. In samples AC05 and AC10, the peaks of illite and kaolinite phases remain unchanged under the oriented conditions and saturated with glycol. In AC10, there is a small displacement of the montmorillonite peak when saturated with glycol. In the heated state (550 °C), the kaolinite phase peaks disappear. This behavior was already expected due to the dehydroxylation of kaolinite at temperatures around ~500 °C [16,17].

Table 1a,b show the chemical composition of the sieved clays (<75 μm) (coarse fraction) and clay fraction separated by sedimentation (<2 μm). For the coarse fraction (Table 1a), all samples showed high contents of SiO_2_ (54–87 wt%) and contents of Al_2_O_3_ that varied between 7–15 wt%. Regarding Fe_2_O_3_, all samples showed Fe_2_O_3_ content in the order of 1–6 wt%. [18]. Iron oxide, present in clays, acts as a flux and is responsible for the red (hematite) or yellow (goethite) color after firing in an oxidizing atmosphere [19,20]. However, it is recommended that the Fe_2_O_3_ content does not exceed 10% because when the material is fired in a low oxygen atmosphere, the high percentages of Fe_2_O_3_ can lead to the formation of the effect called “black core”. The black core produces a bloating on the ceramic piece, compromising the mechanical performance. As already mentioned, all the investigated clays have Fe_2_O_3_ contents lower than 10% and are in accordance with the recommended limit for the production of red ceramic products [21].

Additionally, in Table 1a, alkaline and alkaline earth oxides were detected in a lower percentage than the oxides mentioned above. The alkaline (NaO + K_2_O) concentration was not higher than 5.5 wt%, while the alkaline earth (MgO + CaO) reached the maximum value of 4 wt%. The exception was the sample AC08, which presented CaO contents values around 9 wt%, which is related to the presence of the mineral calcite (CaCO_3_) [22,23,24], as confirmed by X-ray diffraction analyses. During firing, calcite decomposition (CaO + CO_2_) occurs, and the CaO formed can cause an expansion in the ceramic material and consequently the appearance of cracks. Cracks compromise the mechanical strength of the material as they act as stress concentrators. However, depending on the firing temperature, CaO can react with amorphous SiO_2_ and form wollastonite (CaSiO_3_), providing an increase in mechanical strength [21,25].

The K_2_O contents vary from 1–5 wt%. Potassium is associated with illite and feldspars and is an essential element in ceramic formulations because it acts as a flux. The other oxides (TiO_2_, P_2_O_3_), in all samples, are present in contents below 1 wt%. In all samples, the loss on ignition (LOI) is in the range of 3–8 wt%, and the SiO_2_/Al_2_O_3_ ratio in weight varies between 4–11 wt% in the coarse fraction. Table 1b summarizes the chemical analysis of the fine clay fraction separated by sedimentation (<2 μm). The clay fraction samples showed LOI values in the range of 12.33 to 18.40%. Such values are probably associated with coordinated and adsorbed water losses, clay minerals dehydroxylation, and elimination of organic matter.

**Figure 6:**
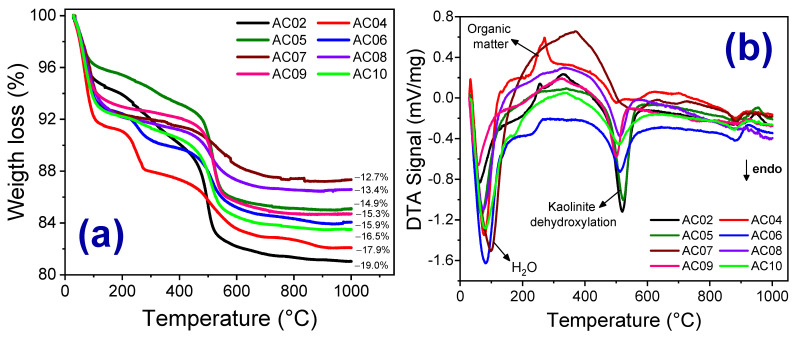
TG (**a**) and DTA (**b**) curves of the samples AC02, AC04, AC05, AC06, AC07, AC08, AC09, and AC10, fine fraction.

Figure 6a,b show the TG (Figure 6a) and DTA (Figure 6b) curves of the clay fractions (AC02, AC04, AC05, AC06, AC07, AC08, AC09, and AC10). In all samples, the total mass loss was less than 19%, similar to the results obtained by X-ray Fluorescence presented in Table 1b. Thermal analyses (TG and DTA) were performed on the clay fraction (<2 µm) to avoid the possible overlapping among the endothermic peaks corresponding to the structural water of kaolinite (~450–600 °C) and the transition of quartz-α to quartz-β (~570 °C).

In general, the decomposition of clays occurred in four events; namely: (i) between 50–150 °C, there is an endothermic peak in all samples, which can be attributed to the evaporation of water adsorbed to the external surface of the particles of the clay minerals present [12,26,27,28]; (ii) between 200–425 °C, an exothermic peak is observed in AC02, AC04, AC07, and AC09; which corresponds to the combustion of organic matter present in the raw material, which is usually associated with sediment illite [28]; (iii) between 425–625 °C, samples AC02, AC05 and AC09 shown an endothermic peak, which is associated with the decomposition of kaolinite and the formation of metakaolinite [26,27]. In AC04, AC07, and AC10 samples, the same thermal event occurs with less intensity and can be attributed to the presence of illite and clay minerals from the smectite group [28]. Figure 4a shows that AC04 is mainly made up of illite clay minerals; (iv) between 850–960 °C, the event can be endothermic or exothermic, depending on the clay composition. In AC04, AC06, AC07, and AC10, the event is endothermic due to the presence of micas (illite) and the destruction of the crystalline lattice of the smectite clays [29,30], since there was no change significant in the mass. However, AC02, AC05, AC08, and AC09 show an exothermic event (930–960 °C), which can be attributed to the formation of spinel [26,27].

Table 2 summarizes all the thermal events identified in each investigated sample. In general, the samples showed a similar thermal profile. However, it should be noted that samples AC03 presented an endothermic event in the temperature range of 200–425 °C. Such an endothermic event may be associated with the removal of crystallization water, common in goethite. However, more than one event may occur simultaneously, such as decomposition of goethite and hematite formation [28].

### 3.2. Particle Size Distribution and Plastic Behavior of the Clay Raw Materials

The particle size distribution and Atterberg limits for all samples investigated in this work are listed in Table 3. It was observed that most of the samples have a larger fraction of particles with diameters less than 53 µm, with 42–97% of passing volume. According to Table 3, it can be said that samples AC02 have a liquidity limit (LL) of around 36%, while in samples AC03, AC04, AC05, AC06, AC07, AC08, AC09, and AC10, this property is between 40–52%. An analysis of the plasticity limit values (PL) reveals that all samples, except AC07, presented values above 20%. Based on these results, it was possible to calculate the values of plasticity index (PI) of the studied samples, which showed values greater than 15%, except for AC10, which has PI values below 14% [31].

**Figure 7:**
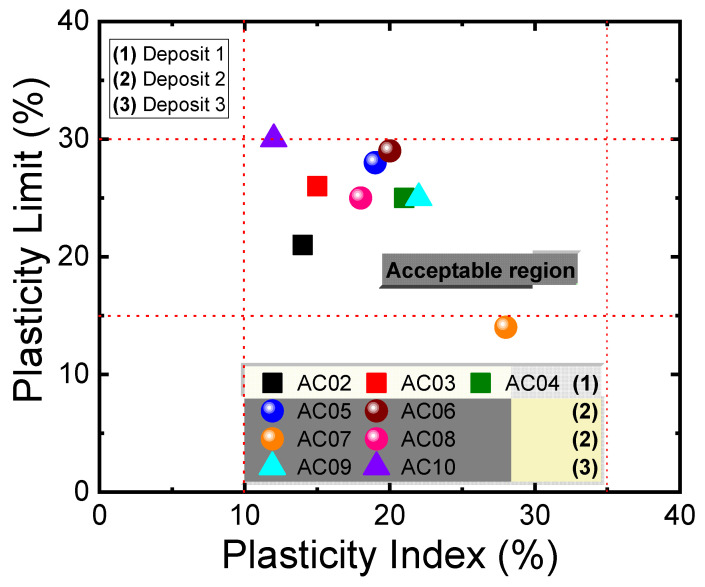
Values obtained in the Atterberg tests and the range of acceptable values for PI and PL [12,28,32,33].

There is no consensus in the literature regarding the appropriate values to express the Atterberg Limits in masses or clays used in red or structural ceramics since other parameters influence the plastic behavior (granulometry, mineral composition, processing method, etc.). In Table 4, values found in the literature for the Atterberg Limits recommended by several authors are shown [12,28,32,33]. Figure 7 compares the values of the plasticity limit obtained in this work with values from the literature. Most of the investigated samples showed acceptable values of PL and PI, indicating that these materials can be used in the red or structural ceramic industry [12,28]. It is important to highlight that, from a technological viewpoint, the PI value indicates the minimum water content to be added to a ceramic mass so that it can be confirmed by the extrusion process [34]. However, materials with high plasticity are not always desired in the ceramic sector. They can impair the pieces’ drying process, causing excessive contraction with the formation of cracks and dimensional changes. Such behavior leads to higher energy expenditure and, consequently, lower process yield [35]. In this context, it is recommended to use several clays with different granulometry in the ceramic masses to obtain an ideal formulation to avoid these problems in the process.

### 3.3. Cation Exchange Capacity (CEC) and Specific Area (SA) of the Clay Raw Materials

**Figure 8:**
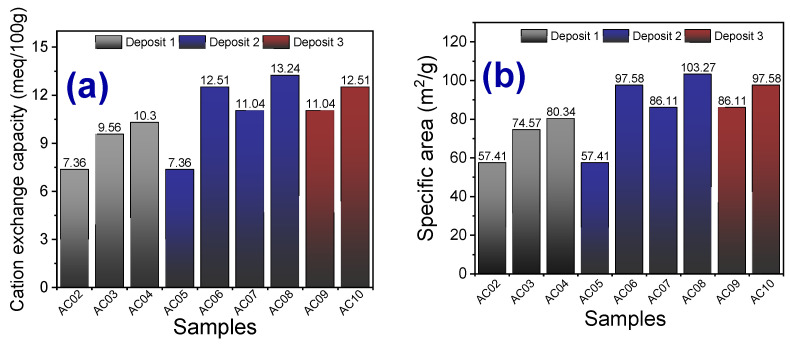
Cation exchange capacity (**a**) and specific areas (**b**) measured from the studied clays.

Figure 8a,b show the cation exchange capacity (CEC) values and specific area (SA) measured by the methylene blue staining test. It was observed that the measured CEC values varied between 2–21 meq/100 g, which can be attributed to the mixture of clay minerals of the kaolinite and illite groups [36] (see Figure 4a,b). The values of the specific area showed the same trend as the CEC values; that is, it increased from deposit 1 to deposit 2. The low values of CEC presented by the studied materials confirm the absence of high amounts of montmorillonites in the mineral composition of these clays [37]. Based on the values presented in Figure 8, the studied samples can be considered raw clay material consisting of a mixture of mica/illite, kaolinite, montmorillonite clay minerals with free silica carbonates, laterite, and water that are essential constituents of clays [38].

### 3.4. Linear Shrinkage after Firing, Water Absorption, Bulk Density, Apparent Porosity, and Flexural Strength

Regardless of the composition, all specimens showed a red color typical of compositions containing Fe_2_O_3_. Table 5 shows the results of the tests: linear shrinkage (LS_F_), water absorption (WA), apparent porosity (AP), bulk density (BD), and three-point flexural strength (FS). Negative values LS_F_ were observed for the sample AC04 (−0.70%); this indicates an expansion of this specimen. This expansion can be caused by the gases trapped inside the specimens due to the combustion of organic matter in the clays and decomposition of carbonates to form CaO or MgO, releasing CO_2_ [36]. The rest of the samples showed LS_F_ values between (0–6.64%), with high AP values (19–31%), indicating that there was no good sintering in most samples, which makes it necessary to increase the firing temperature of these materials above 950 °C. The AC06 and AC08 samples were the exceptions. A minimum LS_F_ value can be positive as it reduces cracks and defects in the ceramic process during drying and firing [34].

It is worth mentioning that the AC08 sample presented the largest linear retraction (6.64%) and that the XRD confirmed the presence of calcium carbonate (calcite) in its composition, as well as high amounts of illite that indicate high amounts of K_2_O. K_2_O promotes the formation of a small amount of liquid phase during firing, which justifies the high values of BD and FS for the AC08 sample [39]. In processing masses for red ceramics, an increase in illite content with a diameter below 2 µm increases the strength at flexion, as this property depends on the granulometric distribution and mineralogical composition of the clays [40].

Still, in Table 5, the measured water absorption values ranged from 10% to 17%. This value range is in accordance with the maximum recommended value for the manufacture of roof tiles (WA < 20%) and ceramic blocks for structural masonry and for sealing (8% <WA < 22%) [41,42]. In this way, the behavior of the studied samples, because of the water absorption capacity, is coherent and suitable for manufacturing tiles and bricks. The bulk density of the samples varied from 1 to 2 g.cm^−3^^,^ and the apparent porosity varied from 19 to 31%.

**Figure 9:**
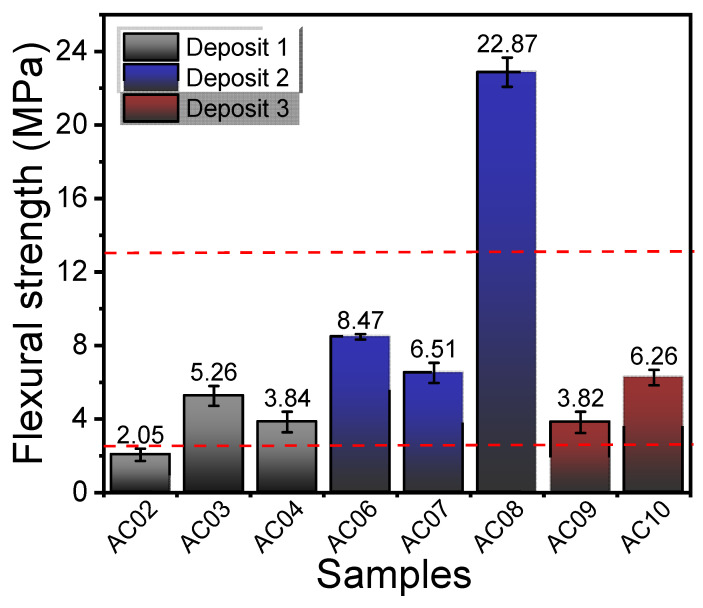
Flexural strength of the specimens after firing at 950 °C by 3 h.

Figure 9 shows FS values for all studied samples. It was observed that the AC03, AC04, AC06, AC07, AC09, and AC10 samples present FS values within the reference range (2.5–13 MPa) recommended for the manufacture of red ceramic materials. On the other hand, the AC02 sample showed a value below the recommended minimum (2.5 MPa). The AC08 sample, on the other hand, showed high FS values above 13 MPa. This behavior can be attributed to the small particle size, as well as the high K_2_O content (5.14 wt%) and the mineral constituents (mica/illite) observed by XRD in this sample. It is known that potassium favors the formation of a liquid phase, increasing mechanical resistance. Based on these values, it is possible to affirm that all the studied clays can be used as raw material for the manufacture of structural ceramic materials; however, it is worth mentioning that all the studied samples present values above 2.5 MPa, which exceeds the minimum values for mechanical resistance of masonry blocks [42].

## 4. Conclusions

Clays from newly discovered deposits located in the city of Caxias-MA (Brazil) were investigated to determine their potential for the manufacture of roof tiles and bricks. The XRD results showed that the investigated clays are mostly made up of mixtures of kaolinite–illite, quartz, and hematite, with the presence of rutile, calcite, and montmorillonite as minor mineral phases. The thermal analysis results (DTA and TG) and cation exchange capacity also indicated the presence of illite and kaolinite. The ceramic specimens produced after firing at 950 °C showed water absorption and flexural strength ranging from 10 to 17% and from 2 to 23 Mpa. The highest flexural strength values were observed for the samples of deposit 2 (AC06, AC07, and AC08). Linear shrinkage tests (LS_F_), water absorption (WA), apparent porosity (AP), bulk density (BD), and three-point flexural strength (FS) indicated that the investigated clays have a high potential for the manufacture of roof tiles and bricks; however, it is necessary to adjust the granulometry of the masses for structural ceramic processing.

## Figures and Tables

**Table 1:** Chemical composition of clays.

**(a) Clays with Coarse Granulometry (<75 µm) (in wt%).**												
	**Samples**	B	**Al_2_O_3_**	**Fe_2_O_3_**	**MgO**	**TiO_2_**	**K_2_O**	**CaO**	**Na_2_O**	**P_2_O_5_**	**SiO_2_/Al_2_O_3_**	**LOI**
Deposit 1	AC02	69.07	14.41	6.20	1.00	0.71	1.00	0.27	0.25	0.05	4.79	6.30
	AC03	72.01	13.82	5.12	0.64	0.63	1.29	0.05	0.11	0.11	5.21	6.11
	AC04	72.55	11.69	3.50	1.54	0.51	3.50	0.10	0.18	0.14	6.20	6.04
Deposit 2	AC05	86.67	8.09	1.00	0.29	0.32	1.03	0.05	0.09	0.05	10.71	2.98
	AC06	71.14	12.83	3.02	1.49	0.69	2.79	0.09	0.20	0.05	5.54	7.89
	AC07	71.04	11.80	3.39	1.59	0.57	3.17	0.37	0.25	0.12	6.02	7.42
	AC08	56.16	13.48	6.22	3.31	0.51	5.14	8.84	0.14	0.17	4.16	5.96
Deposit 3	AC09	70.70	14.18	4.58	1.01	0.77	1.76	0.06	0.19	0.15	4.98	6.70
	AC10	68.70	12.99	3.72	1.77	0.66	3.17	0.27	0.18	0.10	5.28	8.36
(**b**) clays with fine granulometry (<2 µm) (in wt%).												
	**Samples**	**SiO_2_**	**Al_2_O_3_**	**Fe_2_O_3_**	**MgO**	**TiO_2_**	**K_2_O**	**CaO**	**Na_2_O**	**P_2_O_5_**	**SiO_2_/Al_2_O_3_**	**LOI**
Deposit 1	AC02	42.15	27.61	11.52	1.35	0.82	1.90	0.07	0.10	0.10	1.53	13.99
	AC03	46.18	27.62	7.72	1.36	0.82	2.03	0.07	0.15	0.13	1.67	13.74
	AC04	47.20	19.90	6.77	3.12	0.51	4.68	0.33	0.75	0.30	2.37	15.68
Deposit 2	AC05	47.85	29.46	2.48	1.11	0.60	1.33	0.10	0.72	0.11	1.62	15.39
	AC06	48.63	21.26	5.13	2.66	0.62	3.18	0.14	0.57	0.13	2.29	18.40
	AC07	49.22	19.97	6.84	4.13	0.64	4.80	0.66	0.73	0.55	2.46	12.33
	AC08	47.21	17.24	6.32	4.55	0.60	4.62	1.95	0.45	0.12	2.74	16.48
Deposit 3	AC09	45.59	25.34	3.15	2.34	0.65	3.46	0.19	0.67	0.17	1.80	18.39
	AC10	50.71	20.85	5.64	2.70	0.78	3.70	0.35	0.68	0.15	2.43	13.57

**Table 2:** Thermal events present in all the clayey raw materials studied, corresponding to the fine fraction of clay (fraction < 2 μm).

Samples	Thermal Events (°C)					
	50–200 °C	200–425 °C		425–600 °C	850–930 °C	930–960 °C
	Endo	Endo	Exo	Endo	Endo	Exo
AC02	x x	---	x	x x	---	x
AC03	x x	x	---	x x	x	x
AC04	x x	---	x x	x	x x	---
AC05	x	---	---	x x	---	x
AC06	x x	---	x	x x	x	x
AC07	x	---	x	x	x	---
AC08	x	---	---	x	x	---
AC09	x	---	x	x x	x	x x
AC10	x	---	x	x	x x	x
Associated event	Loss of free and adsorbed water	Decomposition of goethite and hematite formation	Combustion of organic matter	Kaolinite dehydroxylation	Destruction of the crystalline lattice of the smectite clays	Formation of spinel

**Table 3:** Particle size distribution and values of the Atterberg limit obtained in the present study.

	Samples	Particle Size Distribution (%)			Atterberg Limits (%)		
		<45 µm	45–75 µm	>75 µm	LL	PL	PI
Deposit 1	AC02	68.9	14.1	17.0	35	21	14
	AC03	78.8	16.2	5.0	41	26	15
	AC04	89.8	9.9	0.3	46	25	21
Deposit 2	AC05	42.3	57.1	0.6	47	28	19
	AC06	97.3	2.4	0.3	49	29	20
	AC07	89.8	7.1	3.1	42	14	28
	AC08	94.1	2.5	3.4	43	25	18
Deposit 3	AC09	88.1	5.4	6.54	47	25	22
	AC10	96.9	3.1	0.0	42	30	12

**Table 4:** Values indicated for plastic behavior found in the literature.

References	PL (%)	PI (%)	Plasticity Level
Dondi [33]	18 ≤ PL ≤ 30	10 ≤ PI ≤ 35	Acceptable
Boussen [32]	20 ≤ PL ≤ 35	15 ≤ PI ≤ 45	Acceptable
Ngun [12]	-	10 ≤ PI ≤ 40	Acceptable
Vieira [28]	20 ≤ PL ≤ 30	10 ≤ PI ≤ 35	Acceptable

**Table 5:** Technological properties of specimens fired at 950 °C for 3 h.

Samples	Physico-Mechanical Properties				
	LS_F_ (%)	WA (%)	AP (%)	BD (g/cm^3^)	FS (MPa)
AC02	0.1 ± 0.2	16.9 ± 0.5	31.2 ± 0.6	1.8 ± 0.1	2.1 ± 0.3
AC03	1.5 ± 0.1	14.3 ± 0.5	27.2 ± 0.6	1.8 ± 0.1	5.3 ± 0.5
AC04	0.0 ± 0.1	12.4 ± 0.5	24.0 ± 0.8	1.8 ± 0.1	3.8 ± 0.6
AC05	-	-	-	-	-
AC06	4.7 ± 0.3	11.6 ± 0.1	23.0 ± 0.4	2.0 ± 0.1	8.5 ± 0.2
AC07	0.7 ± 0.1	13.4 ± 0.5	26.0± 0.9	2.0 ± 0.1	6.5 ± 0.6
AC08	6.6 + 0.3	10.2 ± 0.1	20.4 ± 0.5	2.0 ± 0.1	22.9 ± 0.8
AC09	0.3 ± 0.1	16.2 ± 0.9	29.5 ± 0.7	1.8 ± 0.1	3.8 ± 0.6
AC10	0.2 ± 0.1	11.9 ± 0.1	25.0 ± 0.4	1.9 ± 0.1	6.3 ± 0.4

## Data Availability

The data presented in this study are contained within the article.

## References

[1] da Costa F.P., Fernandes J.V., de Melo L.R.L., Rodrigues A.M., Menezes R.R., de Araújo Neves G. (2021). The potential for natural stones from northeastern brazil to be used in civil construction. Minerals.

[2] CBIC—Brazilian Chamber of the Construction Industry GDP Brazil and Civil Construction. http://www.cbicdados.com.br/home/.

[3] Lee V.-G., Yeh T.-H. (2008). Sintering effects on the development of mechanical properties of fired clay ceramics. Mater. Sci. Eng. A.

[4] Mahmoudi S., Srasra E., Zargouni F. (2008). The use of Tunisian Barremian clay in the traditional ceramic industry: Optimization of ceramic properties. Appl. Clay Sci..

[5] Anicer-National Association of the Ceramic Industry Sector Data. https://www.anicer.com.br/anicer/setor/.

[6] ABCERAM-Brazilian Ceramic Association CERAMIC SEGMENTS (COMPANIES & PRODUCTS). https://abceram.org.br/segmentos-ceramicos/.

[7] de Lima A.G.B., Delgado J.M.P.Q., Nascimento L.P.C., de Lima E.S., de Oliveira V.A.B., Silva A.M., Silva J. (2020). Clay Ceramic Materials: From Fundamentals and Manufacturing to Drying Process Predictions. Transport Processes and Separation Technologies.

[8] Ribeiro J.A.P., Melo F. (2000). Basic Geological Surveys of Brazil. Caxias, Sheet SB.23-X-B. Maranhão State, Brazil. Scale 1:250.000.

[9] Türköz M., Tosun H. (2011). The use of methylene blue test for predicting swell parameters of natural clay soils. Sci. Res. Essays.

[10] Sahin H., Gu F., Lytton R.L. (2015). Development of Soil-Water Characteristic Curve for Flexible Base Materials Using the Methylene Blue Test. J. Mater. Civ. Eng..

[11] ASTM C837-09 (2019). Standard Test Method for Methylene Blue Index of Clay.

[12] Ngun B.K., Mohamad H., Sulaiman S.K., Okada K., Ahmad Z.A. (2011). Some ceramic properties of clays from central Cambodia. Appl. Clay Sci..

[13] ASTM D4318-17e1 (2017). Standard Test Methods for Liquid Limit, Plastic Limit, and Plasticity Index of Soils.

[14] ASTM C674-13 (2018). Standard Test Methods for Flexural Properties of Ceramic Whiteware Materials.

[15] ASTM C20-00 (2015). Standard Test Methods for Apparent Porosity, Water Absorption, Apparent Specific Gravity, and Bulk Density of Burned Refractory Brick and Shapes by Boiling Water.

[16] da Costa F.P., Bezerra I.M.T., Fernandes J.V., Rodrigues A.M., Menezes R.R., Neves G.d.A. (2021). Durability of Sustainable Ceramics Produced by Alkaline Activation of Clay Brick Residue. Sustainability.

[17] Rodrigues A.M., da Costa F.P., Beltrão S.L.D., Fernandes J.V., Menezes R.R., Neves G.d.A. (2021). Development of Eco-Friendly Mortars Produced with Kaolin Processing Waste: Durability Behavior Viewpoint. Sustainability.

[18] Osinubi K.J., Nwaiwu C.M. (2006). Design of Compacted Lateritic Soil Liners and Covers. J. Geotech. Geoenviron. Eng..

[19] da Costa F.P., Morais C.R.d.S., Pinto H.C., Rodrigues A.M. (2020). Microstructure and physico-mechanical properties of Al2O3-doped sustainable glass-ceramic foams. Mater. Chem. Phys..

[20] da Costa F.P., Morais C.R.d.S., Rodrigues A.M. (2020). Sustainable glass-ceramic foams manufactured from waste glass bottles and bentonite. Ceram. Int..

[21] Muñoz V., Morales O., Letelier G., Mendívil G. (2016). Fired clay bricks made by adding wastes: Assessment of the impact on physical, mechanical, and thermal properties. Constr. Build. Mater..

[22] de Figueirêdo J.M.R., da Costa F.P., Fernandes J.V., Rodrigues A.M., Neves G.d.A., Menezes R.R., de Lima Santana L.N. (2020). Development of Scheelite Tailings-Based Ceramic Formulations with the Potential to Manufacture Porcelain Tiles, Semi-Stoneware and Stoneware. Materials.

[23] Fernandes J.V., Guedes D.G., da Costa F.P., Rodrigues A.M., Neves G.d.A., Menezes R.R., Santana L.N.d.L. (2020). Sustainable Ceramic Materials Manufactured from Ceramic Formulations Containing Quartzite and Scheelite Tailings. Sustainability.

[24] Alcântara A.C.S., Beltrão M.S.S., Oliveira H.A., Gimenez I.F., Barreto L.S. (2008). Characterization of ceramic tiles prepared from two clays from Sergipe—Brazil. Appl. Clay Sci..

[25] Muñoz Velasco P., Morales Ortíz M.P., Mendívil Giró M.A., Muñoz Velasco L. (2014). Fired clay bricks manufactured by adding wastes as sustainable construction material—A review. Constr. Build. Mater..

[26] Sakizci M., Alver B.E., Yörükoğullari E. (2009). Thermal behavior and immersion heats of selected clays from Turkey. J. Therm. Anal. Calorim..

[27] Seynou M., Millogo Y., Ouedraogo R., Traoré K., Tirlocq J. (2011). Firing transformations and properties of tiles from a clay from Burkina Faso. Appl. Clay Sci..

[28] Vieira C.M.F., Sánchez R., Monteiro S.N. (2008). Characteristics of clays and properties of building ceramics in the state of Rio de Janeiro, Brazil. Constr. Build. Mater..

[29] Fajnor V.Š., Jesenák K. (1996). Differential thermal analysis of montmorillonite. J. Therm. Anal..

[30] FAJNOR V.S. (1995). KUCHTA Effect of Degradation of Montmorillonite by Vibration Grinding on the DTA Curves in the Range 20–1500 °C. J. Therm. Anal..

[31] Goswami R.K., Singh B. (2005). Influence of fly ash and lime on plasticity characteristics of residual lateritic soil. Proc. Civ. Eng. Improv..

[32] Boussen S., Sghaier D., Chaabani F., Jamoussi B., Bennour A. (2016). Characteristics and industrial application of the Lower Cretaceous clay deposits (Bouhedma Formation), Southeast Tunisia: Potential use for the manufacturing of ceramic tiles and bricks. Appl. Clay Sci..

[33] Dondi M. (1999). Clay materials for ceramic tiles from the Sassuolo District Northern Apennines. Geology, composition and technological properties. Appl. Clay Sci..

[34] Monteiro S.N., Vieira C.M.F. (2004). Influence of firing temperature on the ceramic properties of clays from Campos dos Goytacazes, Brazil. Appl. Clay Sci..

[35] Hajjaji W., Hachani M., Moussi B., Jeridi K., Medhioub M., López-Galindo A., Rocha F., Labrincha J.A., Jamoussi F. (2010). Mineralogy and plasticity in clay sediments from north-east Tunisia. J. African Earth Sci..

[36] Meseguer S., Pardo F., Jordan M.M., Sanfeliu T., González I. (2010). Ceramic behaviour of five Chilean clays which can be used in the manufacture of ceramic tile bodies. Appl. Clay Sci..

[37] Baruah B., Mishra M., Bhattacharjee C.R., Nihalani M.C., Mishra S.K., Baruah S.D., Phukan P., Goswameea R.L. (2013). The effect of particle size of clay on the viscosity build up property of mixed metal hydroxides (MMH) in the low solid-drilling mud compositions. Appl. Clay Sci..

[38] Tlili M.F.A., Montacer M.E.G.M. (2008). Mineralogical study of kaolinitic clys from Sidi El Bader in the far north of Tunisia. Appl. Clay Sci. Tunis..

[39] Jeridi K., Hachani M., Hajjaji W., Moussi B., Medhioub M., López-Galindo A., Kooli F., Zargouni F., Labrincha J., Jamoussi F. (2008). Technological behaviour of some Tunisian clays prepared by dry ceramic processing. Clay Miner..

[40] Jordán M.M., Martín-Martín J.D., Sanfeliu T., Gómez-Gras D., de la Fuente C. (2009). Mineralogy and firing transformations of Permo–Triassic clays used in the manufacturing of ceramic tile bodies. Appl. Clay Sci..

[41] ASTM C1167-11 (2017). Standard Specification for Clay Roof Tiles.

[42] ASTM C216-1 (2019). Standard Specification for Facing Brick (Solid Masonry Units Made from Clay or Shale).

